# The first case of isolated facial cutanenous leishmaniasis in a Down syndrome infant: a case report and review of the literature

**DOI:** 10.1186/1757-1626-2-13

**Published:** 2009-01-06

**Authors:** Kotb Abass, Hekma Saad, Alaa A Abd-Elsayed

**Affiliations:** 1 Department of Paediatrics, Maternity and Children's Hospital, Buraidah, Al-Qassim, Kingdom of Saudi Arabia; 2 Department of Public Health and Community Medicine, Faculty of Medicine, Assiut University, Assiut, Egypt

## Abstract

**Background:**

Cutaneous leishmaniasis can be caused by several Leishmania species and is transmitted to human beings and animals by sand flies, Down syndrome is known to cause immunodeficiency that might lead to increase the susceptibility to infection with Leishmania.

Up to our knowledge this is the first case of isolated facial cutaneous leshmaniasis in association with Down syndrome.

**Case presentation:**

A 2 month old Saudi Arabian male infant was admitted in the pediatric ward of maternity and children's hospital, Buraidah, Kingdom of Saudi Arabia for the management of multiple ulcers on his face, two ulcers were big and were surrounded by edema, causing severe disfigurement. This disfigurement caused difficulty in recognizing the facial feature of Down syndrome. The presence of hypotonia, microcephaly, low set ears, bilateral simian creases and wide separation between big toe and other toes directed us to request karyotyping. The result of karyotyping confirmed the diagnosis of Down syndrome.

**Conclusion:**

Children with Down syndrome are immunodeficient, they have been reported to have a complex of immunological alterations which might lead to increased susceptibility to infection.

## Background

Leishmaniasis consists of a group of diverse diseases that affect viscera, skin, and/or mucous membranes with a wide spectrum of clinical activity caused by vector-borne, obligate, intracellular hemoflagellates of the genus leishmania. Three major clinical syndromes are recognized: visceral, cutaneous, and mucocutaneous leishmaniasis. The clinical manifestations of leishmaniasis appear to depend on a complex set of factors, including tropism and virulence of the parasite strain and the susceptibility of the host that may be genetically determined [1].

The true incidence of leishmaniasis is not known. However, the World Health Organization (WHO) has estimated that 350 million individuals are at risk and that the disease is endemic in more than 80 countries. The estimated incidence of visceral leishmaniasis is 500,000 cases per year, and for cutaneous leishmaniasis and mucocutaneous leishmaniasis, it is 1.5 million cases per year. Although the parasite most commonly is transmitted via the bite of the sand fly vector, it may be transmitted also as a result of a laboratory accident, direct person-to-person transmission, and blood transfusion. In addition, there is evidence that it may be transmitted either in utero or during the peripartum period.

Cutaneous leishmaniasis can be caused by several Leishmania species and is transmitted to human beings and animals by sand flies. In endemic countries, diagnosis is often made clinically and, if possible, by microscopic examination of lesion biopsy smears to visually confirm leishmania parasites as the cause. The use of more sophisticated diagnostic techniques that allow for species identification is usually restricted to research or clinical settings in non-endemic countries. The mainstays of cutaneous leishmaniasis treatment are pentavalent antimonials, with new oral and topical treatment alternatives only becoming available within the past few years; a vaccine currently does not exist. Disease prevention and control are difficult because of the complexity of cutaneous leishmaniasis epizoology, and the few options available [2].

## Case presentation

A two months old Saudi Arabian male infant was admitted to the pediatric ward of maternity and children's hospital, Buraidah, Kingdom of Saudi Arabia for the management of multiple ulcers on his face. The ulcers was observed by the parents at the age of one month and progressively increased in size with no history of fever. He was the third child of healthy unrelated boduin Saudi Arabian parents, the mother's age was 35 and the father's age was 40. There was no history of perinatal or postnatal complication. The child was delivered in a tent in the desert where his parents live.

**Figure 1:**
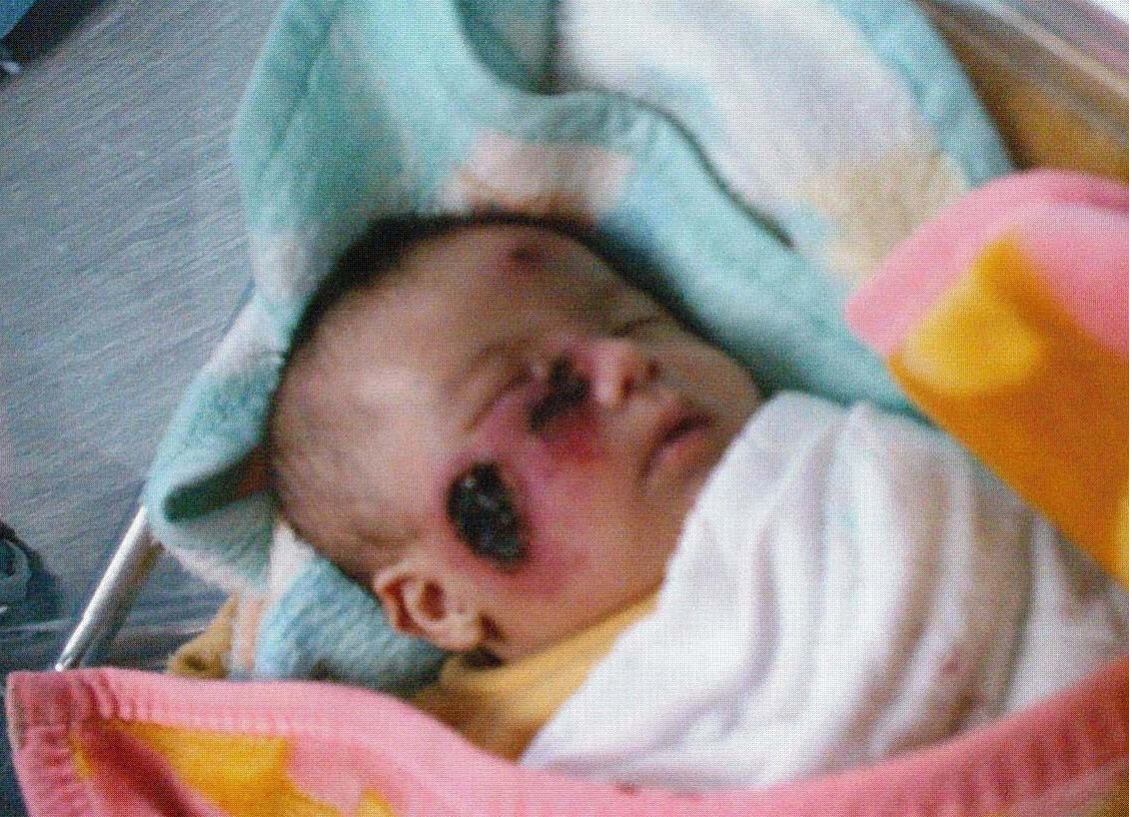
**A child with Down syndrome and Cutaneous Leishmaniasis**.

On physical examination, there was severe disfigurement of the face with 3 crusty ulcers Surrounded by a notable erythematous reaction, the biggest one presents on the right cheek and measures 2 × 2 cm. The second ulcer presents on the right lower eye lid and measures 1 × 1.5 cm. The third ulcer presents on the forehead and measures 0.5 × .5 cm (figure 1). The examination also revealed no lymphadenphathy or hepatosplenomegaly.

On first look to the face of our case, it was difficult to recognize the facial features of Down syndrome because of severe disfigurement of the face, the ulcers were surrounded by extensive edema that caused the eyes to be closed most of the times so the upslanting palpebral fissures which is a very characteristic feature of Down syndrome was not so obvious, in addition the presence of an ulcer near the nasal bridge added more difficulty to recognize the facial features but the presence of hypotonia, flat occiput, short neck, low set ears, short broad hands; clinodactyly of fifth finger; bilateral simian creases of the hands and gap between the first and second toes, directed us to request karyotyping. The result of karyotyping confirmed our suspicion and the diagnosis of Down syndrome was confirmed.

Blood count and immunological study were normal except for the presence of lymphopenia and inverted CD4/CD8 ratio. The baseline erythrocyte sedimentation rate was normal. Liver, kidney, and thyroid function tests (TSH, T3, and T4) were within normal limits. Histopathologic examination of the ulcers' sections stained with hematoxylin and eosin showed a superficial reticular dermis occupied by large pale histiocytes with leishmania. With the Giemsa stain technique multiple leishmania were seen confirming the diagnosis of cutaneous leishmaniasis. After the diagnosis of leishmaniasis, sodium antimony gluconat was administered intravenously along with other antibiotics, the condition started to improve but unfortunately after two weeks of treatment the child was discharged from the hospital under the request and insistence of his parents. The parents were seeking cautery treatment for the ulcers which is a common practice in the Kingdom of Saudi Arabia; the parents refused all medical advices to continue the treatment in the hospital. The child was discharged and was never seen again.

## Discussion

Up to our knowledge this is the first case of isolated facial Cutanenous leshmaniasis affecting the face of a Down syndrome infant. The age of our case is the youngest age affected with cutaneous leishmaniasis in literature.

Ferreli et al reported an atypical leishmaniasis involving the inferior lip of a 57-year-old female with Down's syndrome at the Dermatology Department of Cagliari (Italy) [3].

Children with Down syndrome are immunodeficient [4], they have been reported to have a complex of immunological alterations, supposedly associated with a greatly enhanced susceptibility to infections [5]. Among these immunological changes, a low number of circulating B cells [6], decrease of CD4+ and increase of CD8+ cells with an inverted CD4/CD8 ratio [7], and increased percentage of cells bearing markers associated with NK activity [8] are now well established. Susceptibility to cutaneous leishmaniasis can be greatly influenced by malnutrition [9], immunosuppression (e.g. HIV) [10], host genetic background [11] and less frequently in those who have undergone organ transplant, chemotherapy for malignancy or suffer from immune-mediated disorders. Leishmania and HIV coinfections have been reported in 35 out of the 88 countries in which leishmaniasis is endemic [12].

Cell-mediated immunity appears to be a major factor modulating leishmania infection [13]. Lymphopenia and inverted CD4/CD8 ratio were observed in our case and this can explain the extensive and huge ulcers and affection in the early infancy.

Although cutaneous leishmaniasis does not cause mortality, the cosmetic disfiguration, prolonged period of lesions, great expense of treatment, and side effects of available drugs, it has created many problems [14]. In our case one of the ulcers was located near to lower eye lid, contiguous spread may extend to the conjunctiva, episclera, and cornea, with development of interstitial keratitis, therefore, ocular leishmaniasis is considered potentially a blinding disorder [15].

Previous descriptions of the disease indicated that extremities were more often involved in cutaneous leishmaniasis than the face [16,17]. This finding may be explained by the fact that sand flies usually travel close to the ground where they can easily bite the lower extremities. In addition, sleeping outdoors and the exposure of extremities may encourage sand flies to bite during the night [16]. In our case in addition to sleeping outdoors in the desert and inside tent on ground, the culture traditions in Kingdom of Saudi Arabia is to keep the whole infant's body covered except his face, this can explain why the face is only affected in our case.

The presence of multiple skin lesions on the face of our case may be attributed to the fact that a sand fly, because of its physiologic characteristics, bites the host more than one time, and from every area of the bite, parasites enter the blood [18].

## Conclusion

Children with Down syndrome are immunodeficient, they have been reported to have a complex of immunological alterations which might lead to increased susceptibility to infection.

## Consent

Written informed consent was obtained from the parents of our patient for publication of this case report and the accompanying images.

## Competing interests

The authors declare that they have no competing interests.

## Authors' contributions

KA & HS carried out the patient diagnosis, investigation, follow up and management. AAA-E carried out general coordination, drafting of the manuscript, writing the final manuscript and provided important suggestions. All authors read and approved the final manuscript.
